# Hydrothermal Synthesis of Fluorapatite Coatings over Titanium Implants for Enhanced Osseointegration—An In Vivo Study in the Rabbit

**DOI:** 10.3390/jfb13040241

**Published:** 2022-11-14

**Authors:** Eduardo Santiago, Victor Martin, Bruno Colaço, Maria Helena Fernandes, Catarina Santos, Pedro S. Gomes

**Affiliations:** 1 BoneLab—Laboratory for Bone Metabolism and Regeneration, Faculty of Dental Medicine, U. Porto Rua Dr. Manuel Pereira da Silva, 4200-393 Porto, Portugal; 2 LAQV/REQUIMTE, University of Porto, 4160-007 Porto, Portugal; 3 Animal and Veterinary Research Centre (CECAV), Associate Laboratory for Animal and Veterinary Science—AL4AnimalS, University of Trás-os-Montes and Alto Douro (UTAD), 5000-801 Vila Real, Portugal; 4 EST Setúbal, CDP2T, Instituto Politécnico de Setúbal, Campus IPS, 2910-761 Setúbal, Portugal; 5 Centro Química Estrutural, Instituto Superior Técnico, Universidade de Lisboa, Av. Rovisco Pais, 1049-001 Lisboa, Portugal

**Keywords:** fluorapatite, hydrothermal synthesis, surface functionalization, titanium, osseointegration, microtomography

## Abstract

This work aims at the development and characterization of fluorapatite coatings, innovatively prepared by the hydrothermal method, aiming for enhanced osseointegration of titanium implants. Fluoride-containing coatings were prepared and characterized by scanning and transmission electron microscopy, Fourier-transform infrared spectroscopy—attenuated total reflectance, and X-ray photoelectron spectroscopy. The biological response was characterized by microtomographic evaluation and histomorphometric analysis upon orthotopic implantation in a translational rabbit experimental model. Physic-chemical analysis revealed the inclusion of fluoride in the apatite lattice with fluorapatite formation, associated with the presence of citrate species. The in vivo biological assessment of coated implants revealed an enhanced bone formation process—with increased bone-to-implant contact and bone volume. The attained enhancement of the osteogenic process may be attributable to the conjoined modulatory activity of selected fluoride and citrate levels within the produced coatings. In this regard, the production of fluorapatite coatings with citrate, through the hydrothermal method, entails a promising approach for enhanced osseointegration in implant dentistry and orthopedic applications.

## 1. Introduction

Titanium-based alloys have taken the frontline of reference biomaterials for orthopedic, maxillofacial, and dental therapeutic applications, aiming for bone healing and/or fixation, given the appropriate mechanical, chemical, and biological properties [1]. These include a high strength-to-weight ratio, high yield, and fatigue resistance, as well as an adequate biological response [2]. By and large, the major limitations of titanium-based materials for bone application rely on the potential feeble osseointegration—particularly in aged and disease-affected individuals—which may culminate into interfacial displacement between the implant and the adjacent bone; and the release of metallic cations with potential local and/or systemic toxicity [3,4]. To heighten the implants’ functionality, coating applications—and specifically those with bioactive ceramic materials such as hydroxyapatite (HA)—have been developed, aiming for improved construct stability, long-term functionality, and decreased corrosion [5]. Bioceramic coatings show an effective osteoconduction and potential osteoinductive ability, translated into enhanced bioactivity with the human bone tissue [6]. 

The vast majority of clinically developed bioceramic coating strategies rely on plasma-spraying methodologies. Nevertheless, this coating approach may originate in structural and phase discrepancies—elapsing from the high processing temperature—that create a thick (30–100 μm), highly crystalline, non-uniform coating, and consequently, dissimilar surficial resorption and biofunctionality, as well as a reduction in the interfacial coating-substrate strength [7,8,9]. In the present study, an alternative coating methodology—the hydrothermal method—was used as a simple, scalable, cost-effective, environmentally friendly, and versatile process [9,10]. In addition, it can produce homogeneous coatings on complex-shaped substrates—such as threaded dental implants, with defined chemical composition and crystallinity similar to that of mineral bone tissue [11]. 

In previous reports, the research team established, characterized, and optimized the production of pure-phase HA through this methodology [11]. Aiming for improved bioactivity, the addition of fluoride (F^−^) to the HA lattice, allowing the formation of fluorapatite via hydroxyl substitution, was innovatively assayed in the present study. The fluoride substitution was described to increase the density and reduce the solubility of the bioceramic [12], further improving the biological response within the bone tissue through the release of fluoride ions capable of increasing osteoblastic proliferation and differentiation [13,14]. Notwithstanding, fluoride content needs to be precisely balanced within the materials’ composition, as a high fluoride release may be cytotoxic to bone cells and ultimately impair the bone healing/regeneration process [13,15]. 

Accordingly, this work aims at the preparation and characterization of fluorapatite coatings by the hydrothermal method, with distinct fluoride contents. Coatings were deposited over commercially available titanium implants and, as a proof-of-concept, the biological response of the constructs was evaluated upon surgical implantation within the rabbit proximal tibia and a microtomographic and histomorphometric analysis, at distinct time points.

## 2. Materials and Methods

### 2.1. Preparation and Characterization of Fluorapatite-Coated Titanium Implants 

#### 2.1.1. Implants

Commercially pure titanium Grade 4 implants, AnyRidge^®^ 4 × 7 mm, kindly donated by MegaGen (Seoul, Korea), were used as substrates for coating deposition. Implants were cleaned with water and acetone and placed inside the autoclave until further preparation.

#### 2.1.2. Synthesis of the Hydroxyapatite and Fluorapatite Coatings by the Hydrothermal Method

The hydroxyapatite and fluorapatite coating solutions were produced following the previously described precipitation method [11]. Briefly, a 0.6 M aqueous solution of citric acid (C_6_H_8_O_7_·H_2_O, 99.5%), with a pH of 8.0, was prepared with ammonium solution (NH_4_, 25%). Then, a 0.2 M solution of calcium nitrate ((CaNO_3_)_2_·4H_2_O, 99%) was added to the citric acid solution (solution A). Finally, a 0.2 M, 0.1 M, or 0.01 M solution of ammonium hydrogen phosphate ((NH_4_)_2_HPO_4_) was added, dropwise, to solution A, together with a 0.2 M, 0.1 M, or 0.01 M solution of ammonium fluoride, to obtain the hydroxyapatite coating and two coating solutions with different concentrations of F ions. Finally, the prepared solutions were immediately transferred to a Teflon vessel and placed in the autoclave. The sealed autoclave was set up to 180 °C for 4 h; and the coated implants were named HA, F 0.1, and F 0.01, respectively.

#### 2.1.3. Physical and Chemical Characterization

The morphology of the developed coatings was evaluated using a scanning electron microscope JEOL-JSM7001F, at an operating voltage of 20 kV. The chemical composition of the coatings was determined using an X-ray energy dispersive spectrometer (EDS) analysis. The particle size of the F 0.1 coating was studied using a Transmission Electron Microscope (TEM) (Hitachi H-8100-NA with an acceleration voltage of 200 kV). Before imaging, F 0.1 coating particles were detached from the titanium substrate and dispersed in ethanol. Then, the suspension particles were placed on the carbon-coated copper grid and dried at room temperature. Attenuated total reflectance (FTIR-ATR) spectroscopy using a Nicolet (Thermo Electron) was used to characterize the functional groups and chemical composition of the HA, F 0.01, and F 0.1 coating over a range of 650–4000 cm^−1^ and a resolution of 8 cm^−1^. X-ray photoelectron spectroscopy (XPS; Kratos Axis Ultra HSA, Aluminum mono, Eo = 15 kV (90 W) 1 eV per step in a 300 μm × 700 μm area) was used for fluorine, calcium, and phosphorus content analysis at the surface of the F 0.1 coating.

### 2.2. Biological Characterization—In Vivo Response to Bone Implantation

#### 2.2.1. Animals

In this study, 8 male New Zealand white rabbits (*Oryctolagus cuniculus*) weighing 2.9 ± 0.32 kg were acquired from a certified vendor. The sample size was calculated a priori using G power software (v.3.1.9.6) with the following parameters: significance level (α) was set at 0.05, statistical power (1-β) was set at 0.8, and the effect size (F) was set as at 0.5. All procedures were approved by the local Institutional Animal Care and Use Committee (IACUC), based on standard protocols, under national and European legislation for experimental animal research—European Directive 2010/63/EU.

Animals were acclimatized for 3 weeks before any experimental manipulation, and were housed in environmentally enriched individual cages, in a temperature-, humidity-, and air renewal-controlled room, in a 12 h light-dark cycle. Animals were fed a standard diet (Mucedola 2RB15) and water *ad libitum* and were monitored daily throughout the acclimatization and experimental period. All procedures were conducted in compliance with the ARRIVE guidelines.

#### 2.2.2. Surgical Procedure

Each animal received a total of 6 implants, three on the proximal left tibia and three on the proximal right tibia, which were randomly distributed. Of the 8 animals, half were endorsed for each of the postoperative follow-up periods: 4 and 8 weeks (4 animals per time point, 8 implants per experimental group per time point; allowing the assessment of 8 samples for each group per time point—n = 8) [16].

Before the surgical implantation, animals were pre-medicated with intramuscular injections of 1 mg/kg midazolam. Buprenorphine (0.03 mg/kg), administered subcutaneously, was used for analgesia and continued for 5 days. General anesthesia was achieved upon the intraperitoneal administration of 25 mg/kg ketamine and 5 mg/kg xylazine. Throughout the surgical procedure, sterile saline was administered at 10 mL/kg/h while animals were maintained on a heated surface and carbomer eye gel was administered to prevent ocular lesions. O_2_ was administered by a facial mask throughout the surgical procedure. 

**Figure 1:**
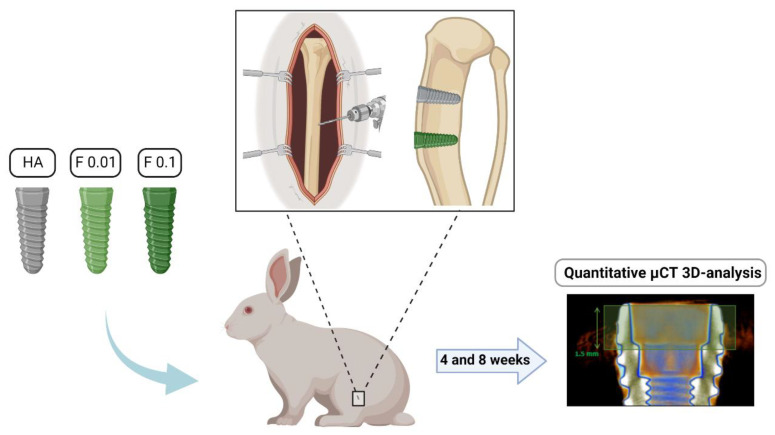
Study design overview.

Following the validation of the anesthetic plane, trichotomy was conducted on both legs that were aseptically prepared for surgery upon chlorhexidine disinfection. Mepivacaine 3% (Scandinibsa, Inibsa) was infiltrated around the incision area and an anteromedial approach to the proximal tibia was conducted. Briefly, a 4 cm full-thickness incision was conducted and upon careful periosteum elevation, the tibial bone surface was exposed. Bone drilling protocol was established as recommended by the manufacturer—lance drill, followed by 2.0, 2.8, 3.2, and 3.8 mm in diameter drills, marked at 7 mm, with the recommended torque values. Implants were placed using the handpiece connector, at a 30 Ncm torque. The soft tissues were then closed in layers with absorbable sutures. During the postoperative recovery, animals were allowed to move freely and were routinely monitored for behavioral and physiological alterations. The biological response to the placed implants was evaluated through microtomography, focusing on the bone formation process in the vicinity of the implants (Figure 1). 

#### 2.2.3. Microtomographic Evaluation

At 4 and 8 weeks upon implantation, animals were euthanized. Tibiae were dissected, fixed in alcohol, and scanned using a Skyscan 1276 system (Bruker, Kontich, Belgium) at 100 kV, 200 uA, using an aluminum/copper filter and a resolution of 10 μm. The scans were performed with a 360° rotation, setting a rotation step of 0.2° and a framing averaging of eight. 

The reconstruction of the obtained projection images was performed with the NRecon software (Bruker, version 1.7.4.2) with fixing parameters, such as bean hardening (16%), ring artifact reduction (0), and minimum/maximum for CS to image conversion of 0.0 to 0.07. Subsequently, implants were aligned along the coronal axis and isolated from each other using DataViewer software (Bruker, version 1.5.6.3). Three-dimensional images were obtained using CTVox software (Bruker, version 3.3.0).

Morphological analysis of the bone around implants was performed using the CTAnalyser software (Bruker, version 1.17.7.2) following the guidelines from Bruker [17]. Briefly, an anatomical reference was selected in the upper portion of the implant, and a fixed height of 1.5 mm was set. Then, the implants were isolated from the bone and other anatomical structures by the binary selection, and a ring of 20 pixels of thickness was drawn around the implant frame, to define the region of interest (ROI). Finally, the images were reloaded and binary thresholding was set to isolate the implant and the bone from the rest of the anatomical structures. The defined bone inside the ROI was analyzed three-dimensionally (bone volume (BV), bone volume fraction (BV/TV), bone surface (BS)), as well as in a 2D approach (bone-to-implant contact, calculated as the percent intersection surface (TIS/TS)—the ratio between total intersection surface (TIS), and total surface (TS)). 

### 2.3. Statistical Analysis

Statistical analysis was conducted on the SPSS software (SPSS Statistics 27, Chicago, IL, USA). Quantitative data are expressed as mean ± standard deviation (SD). The Kruskal-Wallis nonparametric test was used and differences between groups were considered to be significant for *p* < 0.05.

## 3. Results and Discussion

### 3.1. Coating Preparation and Characterization

**Figure 2:**
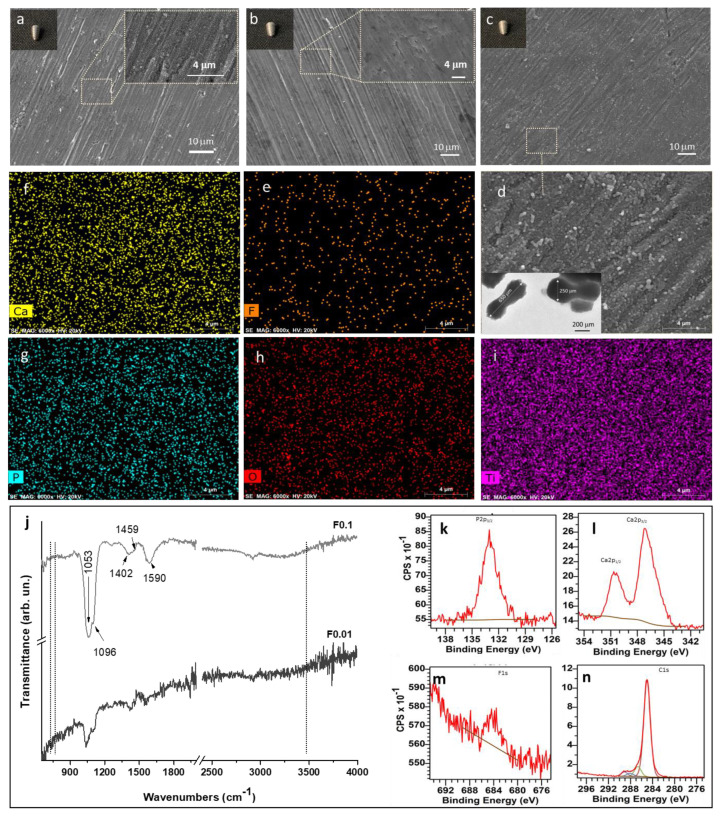
SEM images of the surfaces of the HA (**a**), F 0.01 (**b**), and F 0.1 (**c**) coatings obtained by the hydrothermal method. Magnified SEM image of F 0.1 (corresponding to the square area in (**c**)) and TEM image of F 0.1 coating particles (inset) (**d**). EDS elemental mapping images for F (**e**), Ca (**f**), P (**g**), O (**h**), and Ti (**i**) of the F 0.1 coating surface. ATR-FTIR spectra of the F 0.01 and F 0.1 coatings (**j**). XPS of the F 0.1 coating: (**k**) P 2p, (**l**) Ca 2p, (**m**) F 1s, (**n**) C 1s.

Figure 2 shows the morphology of HA-, F 0.01-, and F 0.1-coated titanium implants and an overall view of the uncoated implant is presented in (Figure S1). The HA coating presents a typical rod-like morphology (Figure 2a) [11]. On the other hand, F 0.01 reveals a uniform F-distributed coating with a “mud-like” morphology, completely covering the titanium surface, without evidence of porosity or discontinuities (Figure 2b). Comparatively, a morphology change was observed on the surface of F 0.1 (Figure 2c), in which individual and aggregated F particles were observed in a homogeneous “dumbbell-like” morphology, further aligned parallelly to the substrate surface (Figure 2d). The average length of the “dumbbell-like” particles determined by TEM was around 650 ± 20 nm by 250 ± 10 nm (inset Figure 2d). The observed morphology change can be attributed to a fractal growth of the fluorapatite particles, caused by the large dipolar field along the c axis of fluorapatite, provided by the presence of citrate ions in the precipitating medium [18,19]. Considering the obtained results, it is reasonable to surmise that citrate molecules have a strong interaction with the fluorapatite particles’ surface, and conditionate the final morphology of the coating. Moreover, it is known that fluoride ions have a higher affinity to occupy positions on the hydroxyapatite lattice in comparison to hydroxyl ions, which enhances thermodynamic stability and decreases the solubility and degradation of the coating. These modifications are expected to lead to more gradual coating resorption, with the added reported benefit of increasing the differentiation behavior of osteoblastic cells and stimulating bone growth, when compared to HA coatings [12,20,21,22]. 

For a detailed elemental characterization, EDS mapping analyses were performed on the F 0.1 coating (Figure 2e–i) and EDS analyses on F 0.01 and HA coating. The obtained results reveal that fluorine (F) (Figure 2e), calcium (Ca) (Figure 2f), phosphorus (P) (Figure 2g), oxygen (O) (Figure 2h), and titanium (Ti) (Figure 2i) were homogeneously distributed over the implant surface. The relative fluoride content of each of the two coatings (F 0.01 and F 0.1), measured by EDS, revealed that the atomic percentage of fluorine ions increase from 0.7% on the F 0.01 coating, to 5.08% on the F 0.1. This result indicates the successful incorporation of F into both coatings. In principle, fluoride ions should be incorporated through the ionic substitution of hydroxyl ions [23].

Based on the previous hypothesis, ATR-FTIR analyses were performed. The notable typical absorption bands at 1053 and 1096 cm^−1^ assigned to stretching vibration of the phosphate groups [18,24] were detected in the ATR-FTIR spectrum of F 0.01 and F 0.1 (Figure 2j). The absence of the characteristic hydroxyl bands at 632 cm^−1^, 3571 cm^−1^, and the presence of one additional band (due to the high concentration of fluorine at ~740 cm^−1^, usually attributed to the libration mode of the OH group connected with a fluoride ion by a hydrogen bond [12,25]) suggests that the formed coating is composed by fluorapatite [26,27,28]. In addition, the absorption peaks at 1402 and 1590 cm^−1^, and a small absorption peak at 1459 cm^−1^, observed in the F 0.1 spectrum revealed the presence of carboxylate groups in the F coating, most probably coming from the citrate species [11]. In addition, according to ATR-FTIR results (Figure 2j), the intensity of carboxylate and phosphates absorption peaks was increased by the F^−^ incorporation into the coating. In our previous results [11], it was found that only a weak band associated with carboxylic groups was observed on the HA coating, while for F-containing coatings, three bands were observed, which confirms that the coordination mode of the citrate species with the particles present in the titanium surface is dependent on the ionic composition of the precipitant medium. In this specific system, the presence of fluoride ions modifies the configuration of citrate species adsorbed, which could have a strong impact on the biological response [29]. Moreover, according to the literature, the presence of citrate in the precipitation medium is expected to enhance the substitution of OH^−^ by F^−^ and accelerate the crystallization process [22]. 

To validate the presence of the fingerprint peak of the fluorapatite structure, XPS analyses were performed and the results are shown in Figure 2k–n. It can be observed that all characteristic peaks of fluorapatite, P2p, and Ca2p, at 133.2 and 347.2 eV, respectively [30], were detected in the F 0.1 coating. Looking in detail, it can be observed one additional small peak at ~684.3 eV, belonging to F1s, indicating that F^−^ ions were incorporated into the fluorapatite lattice structure [31,32]. The F/Ca ratio, calculated directly from the XPS data, was F/Ca = 0.182. In stoichiometric fluorapatite Ca_10_(PO_4_)_6_F_2_, the F/Ca ratio should be, at maximum, 2:10 = 0.2. Considering the obtained ratio, it can be suggested that the produced fluorapatite is close to stoichiometry. Furthermore, looking at the calculated Ca/P ratio (~1.83), it turns out that the ratio is higher than the stoichiometric value Ca/P ratio = 1.67 [31], which may be due to the presence of citrate species on the F-containing coatings. The presence of the citrate species was confirmed by the three fitted peaks, corresponding to C1s photoelectrons from carbon bonded to other carbon and/or hydrogen atoms, carbon singly bonded to oxygen, and carbon in a carboxylate/carboxylic group [11]. Overall, from ATR-FTIR and XPS results, it can be confirmed that the developed F 0.1 coating is composed of fluorapatite. 

### 3.2. Biological Evaluation

The biological characterization of the developed fluorapatite coatings was assayed in an in vivo translational model of the orthotopic implant placement, within the rabbit’s proximal tibia. The rabbit has been a popular choice for the evaluation of biomaterials’ biological response, reaching up to around one-third of the published literature on dental implant-related research [33]. Rabbits reach skeletal maturity at around 6 months of age, and given the fast bone turnover, allow an early evaluation of the bone tissue response [34]. Tibial implantation permits an adequate bone volume for the surgical placement of up to three clinically-relevant implants per side, within the range of 3 to 4 mm diameter and length up to 10 mm, allowing the use of routine characterization techniques to access osseointegration [35,36]. The thick cortical bone—broadly responsible for the primary fixation of the implants—also establishes a favorable environment for the early evaluation of the bone-to-implant interface [34].

The biological response to the developed implant-coated constructs was characterized through microtomography. This technique allows a nondestructive 3D imaging and morphometric analysis of the bone tissue with a very high resolution. Attained datasets can be used to reconstruct the implant and neighboring bone tissue, further characterizing tissue parameters in a given region of interest [37,38,39]. This allows the feasible analysis of bone tissue 3D parameters (e.g., bone volume (BV), bone volume ratio (BV/TV)), and 2D parameters (e.g., bone surface (BS)). In addition, information on the interaction between the implant and the bone tissue (e.g., bone-to-implant contact (BIC)), can also be estimated given proper data processing to minimize titanium-dependent imaging artifacts [40]. 

**Figure 3:**
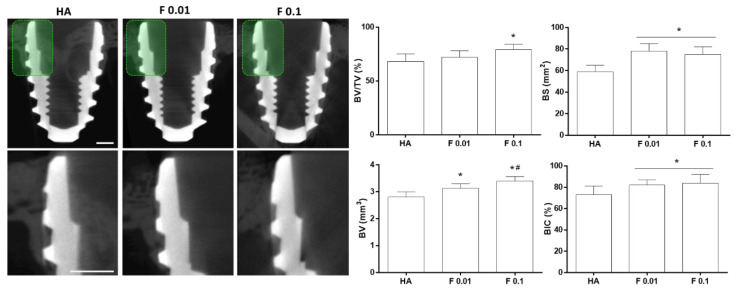
Bi-dimensional microtomographic images (left) and histomorphometric data of the coated constructs—HA, F 0.01, and F 0.1, at the 4 weeks timepoint. Scale bars correspond to 1 mm. *p* < 0.05; * significantly different from control; # significantly different from the other experimental group.

Biological outcomes were evaluated at two time points, 4 and 8 weeks. All animals recovered adequately during the postoperative period without any complications. At euthanasia, no signs of clinical alterations (i.e., ulceration, inflammation, infection, or abnormal tissue formation) were disclosed within the surgical area, with implants remaining integrated in situ. At 4 weeks, sectional reconstructions of the microtomographic data revealed an established cortical bone structure at the coronal aspect of the implant, with newly formed bone tissue growing along the threads (Figure 3), for all the constructs’ compositions. Quantitative volumetric analysis revealed, as compared to HA (control), a significantly higher BV for F 0.01 and F 0.1, with the latter being significantly higher than that of F 0.01. Additionally, F 0.1 presented a significantly higher BV/TV ratio, as compared to HA and F 0.01. In regard to BS, both fluorapatite compositions presented a significantly increased level, a trend similarly verified for the BIC analysis.

**Figure 4:**
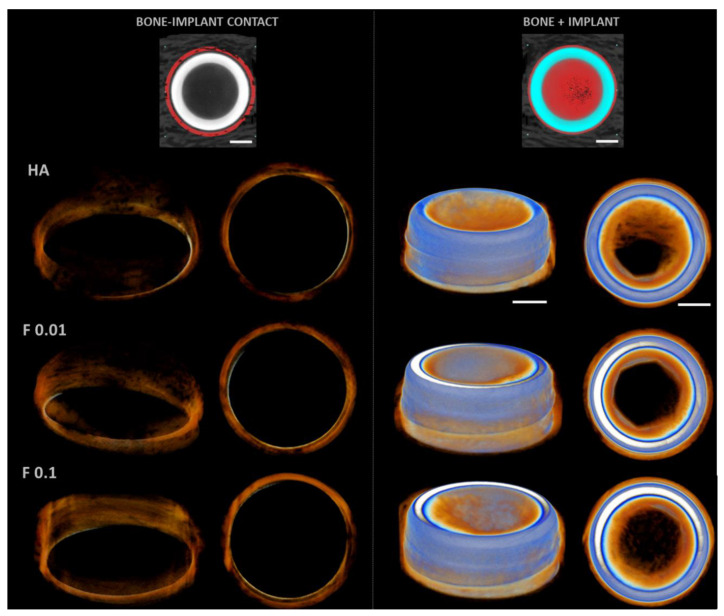
Representative three-dimensional microtomographic reconstructions of the coated constructs—HA, F 0.01, and F 0.1, at the 4 weeks timepoint. Scale bars correspond to 1 mm.

The 3D reconstructions (Figure 4) substantiated the attained morphometric findings, presenting an increased bone volume for fluorapatite-containing substrates, with augmented bone surface and, as well, an increased contact area with the implant. 

**Figure 5:**
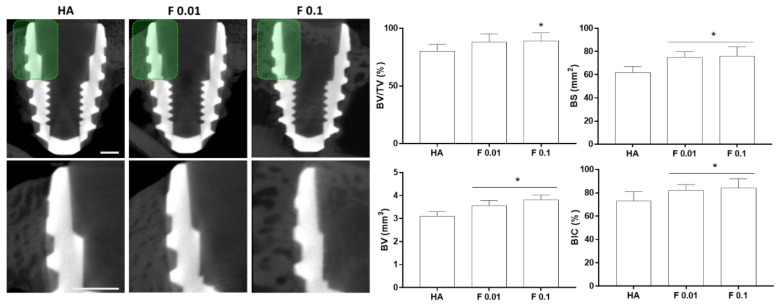
Bi-dimensional microtomographic images (**left**) and histomorphometric data (**right**) of the coated constructs—HA, F 0.01, and F 0.1, at the 8 weeks timepoint. Scale bars correspond to 1 mm. *p* < 0.05; * significantly different from control.

At 8 weeks, a more advanced bone formation process was attained for all conditions, with increased bone levels at the most coronal region of the implants, extending apically along the implant surface (Figure 5). Morphometric data revealed increased levels, as compared to data from the 4 weeks of implantation time (Figure 3). Compared to HA, fluorapatite coatings presented an increased BV and BS, and the F 0.1 formulation further presented an increased BV/TV. Additionally, the BIC was found to be significantly higher in both F 0.01 and F 0.1, despite the absence of differences between conditions.

**Figure 6:**
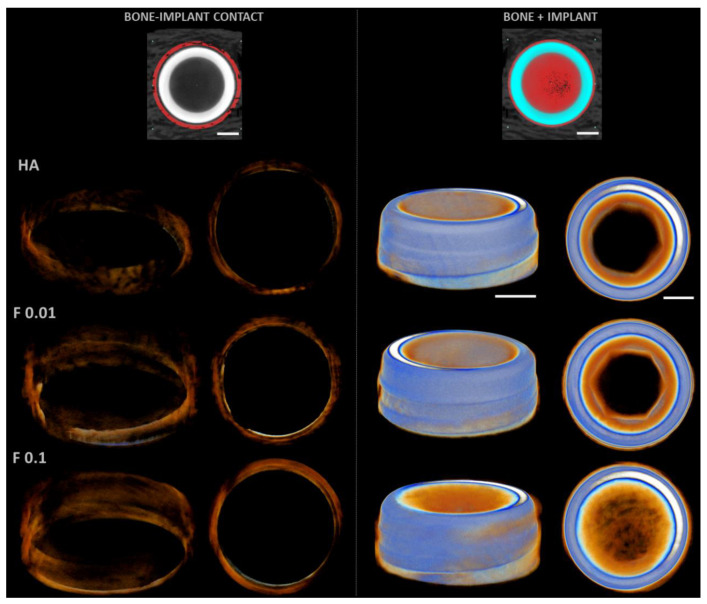
Representative three-dimensional microtomographic reconstructions of the coated constructs—HA, F 0.01, and F 0.1, at the 8 weeks timepoint. Scale bars correspond to 1 mm.

The 3D reconstruction of the peri-implant regions at 8 weeks of healing (Figure 6) corroborates the described findings, with increased bone formation at the coronal region, particularly within fluorapatite coatings, suggesting an increased mineralized tissue volume and increased surficial intersection with the implant surface.

The biological assessment revealed the increased capability of fluorapatite coatings to enhance bone tissue formation in the vicinity of the implant and to increase the bone-to-implant contact, at both 4 and 8 weeks of implantation. F 0.1 coating was found to further induce bone tissue formation at the earliest time point, as compared to F 0.01, in line with the increased F content. Recently, fluoride-containing apatite coatings have become a topic of interest in implantology-related research [41]. Hydroxyapatite has long been considered the bioceramic of choice for bone-related applications, given its biocompatible response, high affinity to the bone tissue, and ability to induce early osseointegration [42]. Clinical applicability has, however, been limited by the reported coating delamination and dubious long-term success [43], associated with the plasma spraying coating technique. The alternative coating strategy currently employed, hydrothermal synthesis, is expected to surpass these limitations given the ability to control crystal structure, crystal morphology, and grain purity of the coating nanoparticles, by adjusting the reaction conditions. In addition, the coating thickness can reach tens of nanometers and uniform coatings can be prepared on complex surface shapes using the hydrothermal method. Furthermore, the prepared calcium phosphate-based coatings have high interfacial bonding strength and density, which can significantly improve the corrosion resistance of metallic substrates [44,45].

In addition, F-containing apatite coatings have demonstrated an enhanced biological response and bioactivity, as compared to hydroxyapatite, within distinct preparation and deposition methodologies [46,47,48]. Higher thermal stability and mechanical properties have also been recognized within F-containing apatites [49,50]. In the present study, F-containing surfaces enhanced bone formation and allowed an increased BIC, with the F 0.1 coating allowing for an enhanced biological outcome. Previous in vitro studies reported an increased osteoblastic proliferation within distinct F-substituted apatites, as compared to non-substituted ones [21]—a process that may be associated with the ability of fluorine to act on relevant cell signaling pathways, as the Jun N-terminal kinase (KNK) and p38 MAPK [51]. Similarly, F-containing substrates were also found to enhance osteoblastic differentiation, thus upregulating the expression of osteogenesis-related markers such as alkaline phosphatase and osteocalcin [52]. Mechanistically, this process may relate to the upregulation of the Wnt signaling pathway via the fluoride-mediated GSK-3β phosphorylation, or via BMP/Smad signaling, also modulated by fluoride [53,54]. On the other hand, F was found to diminish the osteoclastic functionality—either directly through the downregulation of a major transcription factor, NFATc1 [55]; or indirectly, increasing the expression of the osteoprotegerin decoy receptor, inhibiting the osteoblast-mediated osteoclast differentiation [56]. This regulation—a decreased bone resorption conjoined to an increased bone formation, further verified within in vivo models [57], is expected to accelerate the early osseointegration process and consequently, the overall implant success rate.

Nevertheless, in addition to the reported beneficial effects on bone metabolism/regeneration, fluoride may also elicit detrimental effects on bone tissue dynamics, altering cellular functionality and inducing structural damage, as verified in bone fluorosis [58]. The major factor determining fluoride-mediated biological outcomes seems to be the amount of bioavailable F^−^ within the microenvironment [59]. In accordance, both F 0.1 and F 0.01 formulations induced the osteogenic response, with the former outperforming the latter, demonstrating the adequacy of F levels in the coatings’ composition [60]. 

Of additional relevance, the identified citrate species are further expected to tailor the biological outcomes. Citrate is a major component of the bone structure, distributed in two major pools: collagen-associated and HA-associated citrate [61]; and known to enhance the biomineralization process. Citrate also seems to play a chelating activity, binding to important ionic species, such as Mg, Zn, and Ca, constituting a major ionic store in the bone tissue [62]. Exogenous citrate supplementation has been further found to facilitate osteogenesis and cellular commitment of precursor populations, favoring the metabolic changes—switch from aerobic glycolysis to oxidative phosphorylation, needed to meet the increased energetic requirements determined by the osteogenic differentiation [29,62,63]. Citrate molecules may further indirectly induce the expression of osteogenic transcription factors (e.g., RUNX2 and downstream targets) by the stabilization of β-catenin [29,64]. Accordingly, the presence of citrate species on the produced coatings is expected to synergize with F to improve the osteogenic response. 

Limitations of the present study are broadly related to the characteristics of the selected experimental model. While rabbits seem to be appealing models for bone research given the similarity in bone metabolism and Harversian remodeling capability, the anatomical and structural differences and scarcity of biomechanical data on skeletal functionality may hinder direct data translational application [65]. Moreover, future studies should further embrace longer time points of analysis, and embrace orthotopic similarity with functional biomechanical loading, reaching hand, for instance, of the oral implantation in a canine model.

## 4. Conclusions

In the present work, innovative thin ceramic coatings with F^−^ were prepared by the hydrothermal method and deposited over commercial titanium implants. The physicochemical characterization validated the incorporation of F^−^ into the HA lattice through OH^−^ substitution, leading to the formation of fluorapatite, in association with the presence of citrate species. Upon in vivo implantation in the bone tissue, fluorapatite-coated implants presented an enhanced bone formation process at the implant vicinity, with increased bone-to-implant contact, as compared to the control—HA-coated implants. The attained enhancement in osteogenesis is attributable to the conjoined modulatory activity of selected F^−^ and citrate levels, within the produced coatings. The production of fluorapatite coatings with citrate entails a promising approach for enhanced osseointegration in implant dentistry and orthopedic applications.

## Data Availability

The data presented in this study are available on request from the corresponding author.

## Supplementary Materials

The following supporting information can be downloaded at: https://www.mdpi.com/article/10.3390/jfb13040241/s1, Figure S1: SEM image of uncoated titanium implant.
