# Supplementary figures and images for: Hydrothermal Synthesis of Fluorapatite Coatings over Titanium Implants for Enhanced Osseointegration—An In Vivo Study in the Rabbit
Source: J Funct Biomater. 2022 Nov 14;13(4):241. doi: 10.3390/jfb13040241 (PMC9680447; doi:10.3390/jfb13040241)

## Supplementary Materials

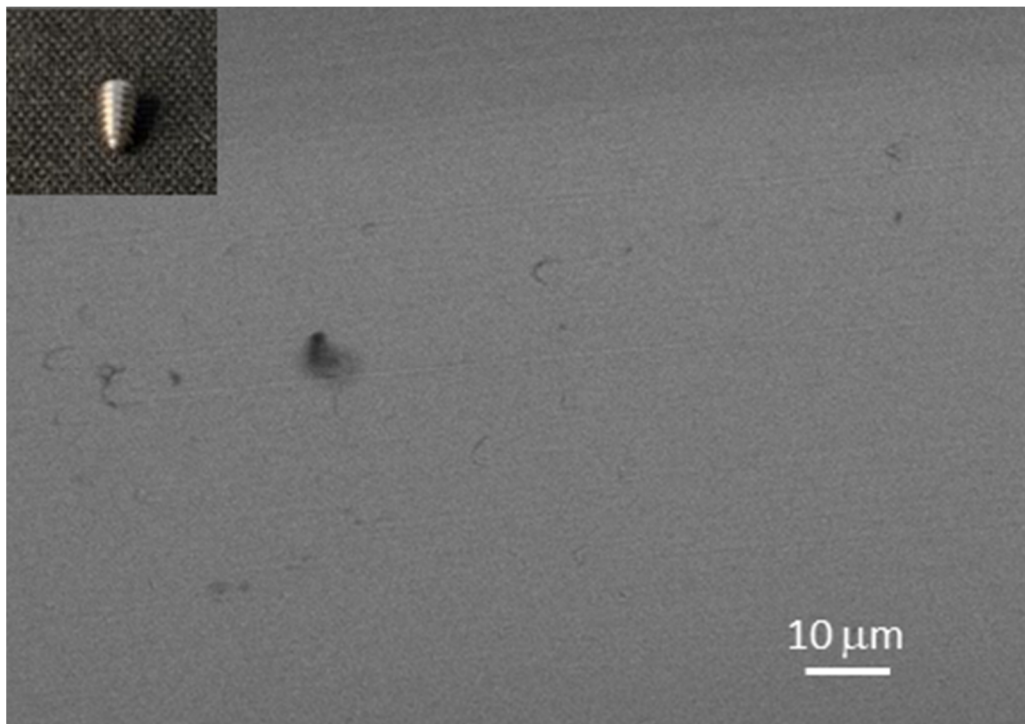

**Figure S1.** SEM image of uncoated titanium implant.

Supplement: Supplementary file 1 [file jfb-13-00241-s001.zip › jfb-2009756-supplementary.pdf]
